# Introduction of microsystems in a level 3 neonatal intensive care unit—an interprofessional approach

**DOI:** 10.1186/s12913-017-1989-6

**Published:** 2017-01-21

**Authors:** Salhab el Helou, Samira Samiee-Zafarghandy, Gerhard Fusch, Muzafar Gani Abdul Wahab, Lynda Aliberti, Ahmad Bakry, Deborah Barnard, Joanne Doucette, Enas el Gouhary, Michael Marrin, Carrie-Lynn Meyer, Amit Mukerji, Anne Nwebube, David Pogorzelski, Edward Pugh, Karen Schattauer, Jay Shah, Sandesh Shivananda, Sumesh Thomas, Jennifer Twiss, Connie Williams, Sourabh Dutta, Christoph Fusch

**Affiliations:** 1 Division of Neonatology, Department of Pediatrics, McMaster University, Hamilton, 1280 Main Street West, Hamilton, ON L8S 4K1 Canada; 2 Department of Pediatrics, General Hospital, Paracelsus Medical School, Nuremberg, Germany

**Keywords:** Microsystem, Neonatal intensive care unit, Organizational structure, Quality care

## Abstract

**Background:**

Growth of neonatal intensive care units in number and size has raised questions towards ability to maintain continuity and quality of care. Structural organization of intensive care units is known as a key element for maintaining the quality of care of these fragile patients. The reconstruction of megaunits of intensive care to smaller care units within a single operational service might help with provision of safe and effective care.

**Methods/Design:**

The clinical team and patient distribution lay out, admission and discharge criteria and interdisciplinary round model was reorganized to follow the microstructure philosophy. A working group met weekly to formulate the implementation planning, to review the adaptation and adjustment process and to ascertain the quality of implementation following the initiation of the microsystem model.

**Discussion:**

In depth examination of microsystem model of care in this study, provides systematic evaluation of this model on variable aspects of health care. The individual projects of this trial can be source of solid evidence for guidance of future decisions on optimized model of care for the critically ill newborns.

**Trial registration:**

ClinicalTrial.gov, NCT02912780. Retrospectively registered on 22 September 2016.

## Background

The medical care of extremely premature infants has made substantial improvements over the past two decades [1]. Advancement in neonatal care along with enhancement of life-saving technologies has resulted in the growth of neonatal intensive care units (NICU) in number and size [1, 2]. Development of large neonatal intensive units with over 40 beds, referred to as mega-units, has raised questions regarding their ability to maintain continuity and quality of care of these fragile patients [3, 4]. Increased rate of adverse events, provider burnout, strained infrastructure and impaired health system’s operation are among the potential risks associated with development of large NICUs.

Structural organization of intensive care units is known as a key element for maintaining the quality of care. The reconstruction of mega-units of intensive care into smaller care units within a single operational service defined by specific patient population, clinical team providers and determined process and purpose might help with provision of safe and effective care [4–6].

The effect of a microsystem model on the organizational structure of NICU and the quality of neonatal care has not been previously described. The microsystem model of neonatal intensive care divides the NICU to designated areas based on the acuity and complexity of the patient care. It also incorporates the patient’s level of complexity of care so that adjusted nurse-to-patient ratio can be applied to staffing distribution [7]. This micro-structure philosophy is believed to facilitate the optimized staffing distribution and quality of care within large NICU’s with varying levels of care needs and staffing expertise.

In spite of increasing awareness of potential benefits of a microsystem model of care, there is no evidence regarding the safety and efficiency of this organizational change in NICUs [3, 5]. The NICU at McMaster Children’s Hospital (MCH) was scheduled for a change from the standard model of care to the microsystem model on the 1st of January 2014. Hence, we aimed to prospectively examine the impact of this organizational change on the quality and efficiency of service delivery on certain key aspects of health care. We hypothesized that the introduction of a microsystem model of care would result in a decreased neonatal morbidity and mortality outcome along with the superior caregiver and health personnel satisfaction.

## Methods/Design

### Setting

#### Patients

The NICU at MCH is a 47-bed, tertiary level facility, with approximate admission rate of 1200 infants per year. It is the level III referral center for a wide catchment area with over 27000 births annually. The rate of admission for infants with birth weight (BW) less than 1250 grams or 30 weeks gestational age (GA) is approximately 150 per year. The patient population of the NICU is composed of patients of high to moderate acuity and chronic complex infants.

#### Clinical team

**Fig. 1:**
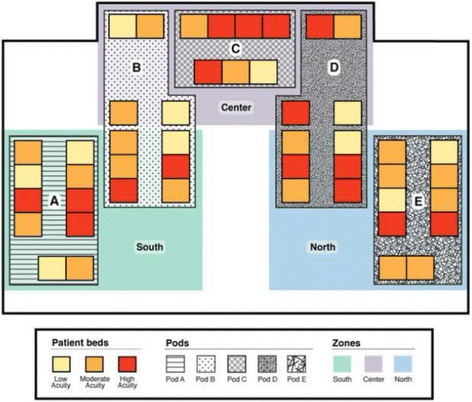
Patient distribution layout in the NICU at MUMC, using standard model of care. South, North and Center zones as labelled

The clinical team at MCH is composed of four lines of service, including three neonatal intensive care service and one consultation service. The usual neonatal intensive care clinical team is composed of a neonatologist, nurse practitioners, a respiratory therapist, nursing staff and neonatal trainees. Nutritionists, occupational therapists and pharmacists attend patient round as per the patient-care need. In the standard model of care, the three neonatal teams were covering the three zones of NICU, which were identical in patient distribution and acuity (Fig. 1). The 12 neonatologists and their randomly assigned medical team members were rotating through the three zones during two-week service periods. In the microsystem model, we divided the 12 neonatologists into three groups, assigning each group to one microsystem zone for a period of 1 year. Each microsystem team was assigned to one microsystem zone (details described later). The four neonatologists in each group were scheduled for rotation through their microsystem team for the one-year period. The consistency of medical trainees and allied health members of each microsystem team was also maintained on a monthly schedule.

The responsibilities of the fourth line of service, includes coverage of labor and delivery and attendance at high-risk deliveries, providing outpatient and inpatient maternal fetal medicine consultations and scheduled teachings. This remained consistent through pre and post implementation of microsystem model. The on call neonatologist covers weekends and night shifts.

#### Working group for implementation of microsystem model of care

The working group consisted of nine health care professionals including three neonatologists, one nurse practitioner, one clinical leader, two managers and two research coordinators, who met weekly between January 2013 until May 2014 to develop the implementation planning. The details of implementation planning are described in the process and pattern section. Following implementation in May 2014, the group continued to meet weekly in order to review the adaptation and adjustment process, ascertain the quality of implementation and address emerging needs of parents or providers.

#### Process and pattern

For the purpose of creating three microsystem units, we established a new layout for patient distribution in NICU, admission and transfer criteria, team structure, interdisciplinary rounds model and a staffing model for each microsystem unit.

#### Patient distribution layout

**Fig. 2:**
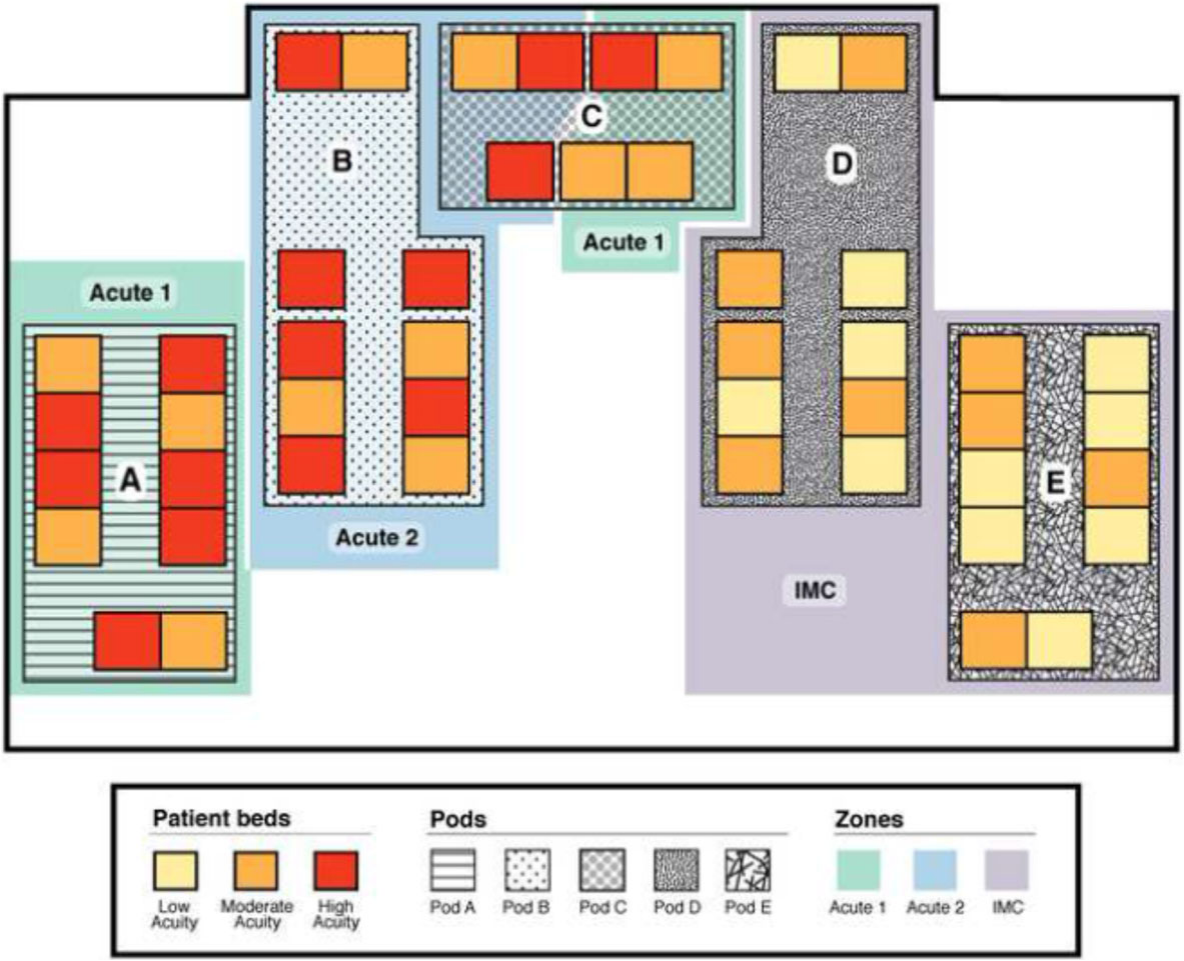
Patient distribution layout in the NICU at MUMC, using Microsystem model 1, based on an anticipated 60% high acuity rate. Moderate to high acuity patients are assigned to Pod A, Pod B and Pod C, Acute 1 and Acute 2 zones as labeled. Low to moderate acuity patients are assigned to Pod D and Pod E, Intermediate Care (IMC) as labeled

**Fig. 3:**
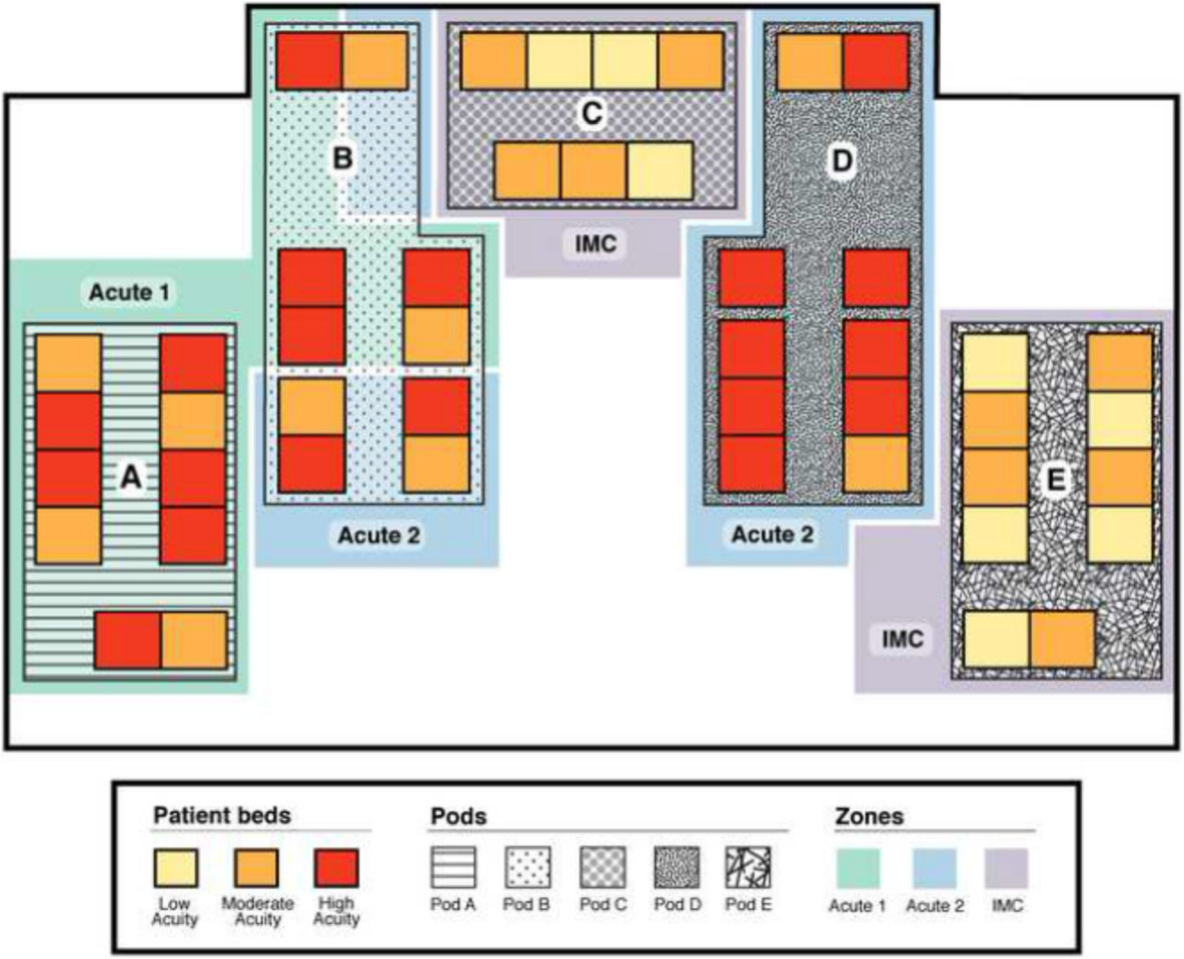
Patient distribution layout in the NICU at MUMC, using Microsystem model 2, based on an anticipated 90% high acuity rate. Moderate to high acuity patients are assigned to Pod A, Pod B, and Pod D, Acute 1 and Acute 2 zones as labeled. Low to moderate acuity patients are assigned to Pod C and Pod E, Intermediate Care (IMC) as labeled

The NICU is comprised of four separate vertical alleys (pods A, B, D and E) and one horizontal alley (pod C). Each of the vertical alleys and the horizontal alley accommodate 10 and seven patient beds respectively. Prior to the introduction of microsystem model, the NICU was following the standard model of care in which the extremely sick infants were interspersed between the less sick ones (Fig. 1). The NICU was divided into three separate zones (south, center and north), consisting of variable distribution of acute and chronic patients. South, center and north zones covered pod A and the front area of B, pod E and front area of D and pod C with the back areas of pod B and D, respectively. Upon change to the microsystem model of care, we assigned pod A, B and C of the NICU to the moderate to high acuity admissions, with anticipated 60% high acuity rate, and the remaining of NICU to the low to moderate acuity patients (Microsystem model 1) (MM1). This pattern remained consistent until 1st of November 2014, when microsystem model 2 (MM2) was introduced. The change from MM1 to MM2 occurred in response to the census and acuity review, which demonstrated close to 90% high acuity rate in the moderate to high acuity zones of NICU and a concurrent increase in the overall admission rate. We thus modified the model to expand the acute zones of NICU and assigned pod A, B and D to the moderate to high acuity admissions and pod C and E to the low to moderate acuity zones (Figs. 2 and 3).

#### Admission and transfer criteria

We defined the criteria for admissions to moderate to acuity acuity zones *a priori* as any of the following: ≤ 30 weeks GA, ≤ 1250 gram BW, on invasive ventilation, continuous positive airway pressure (CPAP) ≥7 cmH2O, fraction inspired Oxygen (FiO2) ≥ 30%, inotropic support, invasive monitoring, defined as an arterial or central venous or intracranial pressure monitoring, hypoxic ischemic encephalopathy, suspected pending cardiorespiratory or neurologic instability, postoperative status and need for ≥ 1:2 nurse-patient ratio of nursing care.

We defined an infant eligible for transfer to the low to moderate acuity zones once the infant met all the following transfer criteria: Current weight ≥ 1000 grams, extubated for ≥ 36 h, CPAP < 7 cmH2O, FiO2 < 30%, not on inotropes, not on invasive monitoring, Feeds > 100mls/kg/day and need for ≤ 1:3 nurse-patient ratio. Any infant who did not meet the moderate to high acuity admission criteria was admitted to the low to moderate acuity pods. Infants with increasing support needs at any point during their hospitalization, meeting any of the admission criteria to the moderate to high acuity areas, required retransfer to these zones. Furthermore, as part of daily bed management process, infants ready to transfer to the low to moderate acuity zones were identified.

#### Interdisciplinary round model

We formulated a new model of structured interdisciplinary bedside rounds for each microsystem unit. We specified start and end times so that presence of multidisciplinary team members could be ascertained. We introduced a new rounds template to standardize report structure and quality. The anticipated structure would allow all team members to exchange perspectives and develop a clear plan. For the purpose of optimizing communication, the bedside nurse initiated the report by providing a short background on the newborn along with highlights of the overnight course. The respiratory therapist, nutritionist and all other involved allied team members contributed to the care by providing relevant information and creating the plan of care. The medical team then formulated the final plan incorporating all inputs.

#### Model calculations on workload and patient transfers

The nurse-patient ratio in the standard model of care was a mandated 1:2 ratio. Upon changing to a microsystem model of care, we changed to an adjusted nurse-patient ratio model, with a maximum 1:2 nurse-patient ratio in moderate to high acuity zones and 1:3.5 nurse-patient ratio in low to moderate acuity zones. To predict the change in nursing workload and number of internal patient transfers as a result of the change to a microsystem model of care, we used patient data from the two-month period of November and December 2013 when the standard model of care was in place. We used Winnipeg Assessment of Neonatal Nursing Needs Tool (WANNNT) to perform a model calculation on nursing workload and patient transfers simulating the proposed microsystem model into existing data [8].

### Purpose

We planned this prospective longitudinal research trial to examine the efficacy of the microsystem model in 4 major areas of health care. We assigned a full time study coordinator to this project so that quality and timely prospective data collection could be reassured. We arranged for weekly meetings of the inter-professional research group specific to development and progress of our research projects. We developed clear prospective plan for data gathering and continuously monitored its accuracy. Data was gathered in the following four areas of health care: 1. patient domain; 2. health care domain; 3. process of care domain; 4. administrative domain. We identified 12 individual projects under the above domains (Table 1). For each outcome we compared the four-month period prior to microsystem implementation to the four-month period after the establishment of the microsystem model. We did not include data during the initial three-month of microsystem introduction (January- March) so that the bias associated with the transition period could be avoided. The results for most administrative and process of care domain projects will be available in spring 2017 with planned publication date of summer 2017. Results of the projects related to long-term outcomes, will be ready in fall 2017 with the following summer as planned publication date. The study protocol was approved by the Hamilton Integrated Research Ethics Board under ID# 12-417. The trial was retrospectively registered at Clinical Trials registry (@clinicaltrials.gov) under ID# NCT02912780.

**Table 1:** Prospective longitudinal research trial on the effect of microsystem model on variable aspects of health care. (last updated on Oct 25th, 2016)

Title	Description	Sample Size^a^	Stage of study
*Effect of microsystem model of care on:*			
Administrative domain			
1. Overarching paper	The objective, framework and purpose of the study	NA	Intervention completed in May 2014, manuscript submitted
2. Prediction of parental stress in NICU	Correlation of salivary cortisol level and questionnaire in predicting stress level	150	Data analysis in progress, poster presented
3. Health care administration pre and post microsystem	Cost, length of stay, patient flow in NICU pre- and post-implementation of microsystem	1 year pre- and 1 year post-implementation	Data analysis in progress
Patient domain			
1. Stress level of HCP, parents and patients in NICU	Salivary cortisol level in HCP, parents and patients	150	Samples analysed, correlation analysis in progress
2. Parental stress in NICU	Parent questionnaire	150	Data analysis in progress
3. Neonatal stress response and long term outcome in infants hospitalized in NICU	Correlation of salivary cortisol level and long-term outcome	60	Data analysis in progress
Health care domain			
4. Short-term outcome of hospitalized infants in NICU	Incidence of ROP, BPD, NEC, sepsis, time on invasive ventilator, blood transfusion, length of stay	200	Data analysis in progress
5. Nutrition and growth of hospitalized infants in NICU	Time of feed initiation, time to full feed, number of NPO days, weight gain	200	Data analysis in progress
6. Long-term outcome of hospitalized infants in NICU	Neurodevelopmental outcome and number of re-hospitalizations in the graduates of NICU	200	Data analysis in progress
Process of care domain			
7. Primary care of hospitalized infants in NICU	Number of changes in the primary health care professionals involved in the care of infants	200	Data analysed, draft of manuscript prepared, submission pending on acceptance of manuscript #1 (overarching paper)
8. Workload of health care professionals	Number of bed movements and nursing workload distribution	1 year pre- and 1 year post-implementation	Data analysed, draft of manuscript prepared, submission pending on acceptance of manuscript #1 (overarching paper)
9. Health environment in NICU	Noise level in NICU environment	1 year pre- and 1 year post-implementation	Data analysed, draft of manuscript prepared, submission pending on acceptance of manuscript #1 (overarching paper)
10. Use of resources in NICU	Laboratory, radiological and microbiology resource use	1 year pre-and 1 year post-implementation	Data analysed, draft of manuscript prepared, submission pending on acceptance of manuscript #1 (overarching paper)

*HCP* Health Care Professional, *NICU* neonatal intensive care unit, *ROP* retinopathy of prematurity, *BPD* bronchopulmonary dysplasia, *NEC* necrotizing enterocolitis, NPO Nil per os

^a^Estimated sample size

### Study status

The 12 individual projects of this trial, are all currently ongoing. Each project is at a different stage, with some still collecting data and some going through data analysis.

## Discussion

In this prospective longitudinal quasi-experimental trial, we aimed to examine the efficiency and safety of establishing a microsystem model in a 47-bed level III NICU. This study is the first study of its kind, which prospectively planned the neonatal microsystem model of care in a large NICU to assess its potential risks and benefits in wide variety of aspects of health care. The prospective design of this trial helped us to address the identified characteristics of a high functioning microsystem through scheduled weekly meeting, inquiring feedback, providing information and continuing education so that essential elements of clinical microsystem could be maintained and positive culture could be fostered [4, 6].

Loss of optimized teamwork and steady relationship, increase rate of serious occurrence reports and loss of communication might be among some potential risks of expansion of NICUs [2]. The growing heterogeneous population of NICUs, present a challenge in providing care that is directed to their specific need. As NICUs enlarge in census, acuity and staff numbers, their structural organization demands prompt reevaluation. As part of adjustment to this growth, the move towards a small, structured patient care unit, along with a stable healthcare team, known as microsystem, appears as a promising option for the optimal provision of critical care in neonates. Using smaller units of care, microsystem optimizes the team members’ partnership, teamwork and communication, all of which can result in improved specialized health care delivery [9, 10]. A model of nursing team microsystems in a 50-bed level III neonatal intensive care unit showed increased family and nurses’ job satisfaction following microsystem implementation [3]. In a study of 23 tertiary-level NICUs, infants admitted to larger NICUs, with higher intensity of resource use, had significantly higher odds of a composite adverse outcome. Authors concluded that there is a need for in-depth examination of NICU microsystem model of care as it might provide the heterogeneous patient population of NICU with an enhanced clinically relevant care [11]. Systematic evaluation of this model, on variable aspects of health care, is thus essential so that solid evidence for guidance of future decisions can be available. Furthermore, it is important to measure the potential impacts so that transition to the new system can be established in the most efficient manner [2, 3, 12, 13].

As the bedside care of extremely sick newborn infants moves toward enhanced standardization, the role of internal organizational factors, in quality of care and patient outcome, becomes further clear. Results of the current trial will be of extreme importance in validating the role of clinical microsystem approach in the meg-aunits of neonatal intensive care.

## Abbreviations

BW: Birth weight
CPAP: Continuous positive airway pressure
FiO2: Fraction inspired oxygen
GA: Gestational age
NICU: Neonatal intensive care unit
